# Early Mobilization and Rehabilitation to Enhance the Functional Performance of a Hemiparesis Patient Following a Subdural and Subarachnoid Hematoma With Pneumocephalus: A Case Report

**DOI:** 10.7759/cureus.51199

**Published:** 2023-12-27

**Authors:** Disha K Rathi, Shraddha S Kochar, Snehal Samal, Akshaya Saklecha

**Affiliations:** 1 Neurophysiotherapy, Ravi Nair Physiotherapy College, Datta Meghe Institute of Higher Education and Research, Wardha, IND

**Keywords:** early mobilization, rehablitation, pneumocephalus, subarachnoid hemorrhage, subdural hematoma

## Abstract

A subdural hematoma (SDH) is a medical condition caused by a violent head trauma in which blood accumulates excessively under the dura mater. It occurs when a blood arterial weak point or brain surface aneurysm ruptures and bleeds. The resulting blood accumulation inside and around the skull raises the pressure on the brain. Pneumocephalus, also known as pneumatocele or intracranial aerogel, refers to air in the ventricular cavities or brain parenchyma's epidural, subdural, or subarachnoid spaces. In most cases, neurotrauma is the primary cause of pneumocephalus, mainly when there are skull base fractures. Here, we present a case study of a 65-year-old male patient diagnosed with left hemiplegia following SDH with subarachnoid hematoma (SAH) and pneumocephalus. The severity of the patient's illness, the medical and surgical care provided, the amount of physiotherapy required to aid recovery, the duration of hospitalization, and the discharge location for patients with acute SAH or SDH vary significantly. The patient underwent physiotherapy rehabilitation, and we report that his lower limb strength improved substantially after the therapy. Therefore, physiotherapy is a critical component of treatment to enhance muscle strength, facilitate early and rapid recovery, and manage the clinical manifestations of the condition.

## Introduction

A subdural hematoma (SDH) is the accumulation of blood between the dura and the arachnoid membranes [1]. It is categorized into three groups, namely, chronic, sub-acute, and acute, with injury to the head being the primary cause of acute SDH [2]. Several factors, including injury, arteriovenous malformation, and anticoagulant substance use, can lead to SDH [3]. The categorization of SDH is based on the size, location, and time elapsed since the initiating event [4]. SDH has a high fatality rate and significant long-term morbidity, with up to 32 percent (%) death rate and a 33% recurrence rate [5]. The underlying cause of SDH is believed to be the cortical veins that bridge the subdural space and empty into the nearby dural sinus, which stretch and tear when the head's velocity suddenly changes, leading to shearing forces and vein rupture. The arachnoid can also tear, resulting in a mixture of cerebrospinal fluid (CSF) and blood in the subdural space [6]. The collection of blood between the arachnoid and pia mater can cause potentially fatal subarachnoid hemorrhages (SAH), with aneurysm rupture being the leading cause of SAH in 85% of cases [7].

The clinical presentation of SDH varies, with the most common symptom being a sudden, severe headache accompanied by meningism, temporary or prolonged unconsciousness, and specific neurological impairments, such as paresis and cranial nerve palsies [8]. Hemorrhagic strokes constitute 20% of all strokes, with 10% being SAH and 10% being intracerebral hemorrhage (ICH). Acute death due to SAH is caused by blood accumulation between the arachnoid and the pia mater. Ten to 14 out of every 100,000 people experience SAH in the United States each year [9]. SAH is more common in women, with a ratio of 3:1, but men are more likely to have it before the age of 40. Aneurysmal rupture is more common as individuals age, peaking in the fifth and sixth decades. The risk of bleeding in individuals with a previously unruptured aneurysm is 1% to 2% per year, with a higher risk for aneurysms more giant than 7 millimeters [10]. Hypertension and cocaine use are linked to ICH and aneurysmal rupture. The presence of air in the ventricular cavities is known as pneumocephalus, which can occur spontaneously, after brain surgery, or as a result of trauma [11]. It can be classified as simple or tension pneumocephalus, and in clinical settings, it can cause headaches, nausea, vomiting, agitation, lightheadedness, and convulsions [12]. Based on the sound of gas input, patients sometimes describe air entry into the cerebral space as a "gurgling" sensation in the head [13].

Our data collection included 1,307 SDH patients, 805 of whom had a history of head trauma, while 502 did not. Physiotherapy plays a critical role in rehabilitation, and an algorithm for step-by-step mobilization was used to enable early mobilization starting from the day following aneurysm repair [14]. Early rehabilitation of patients following SAH is safe and feasible, and higher degrees of mobilization do not increase neurosurgical complications. Instead, early rehabilitation reduces the frequency and severity of cerebral vasospasm following an SAH [15]. The field of neurorehabilitation for traumatic brain injury (TBI) is extensive and complicated, encompassing early patient rehabilitation for those with compromised cognitive, motor, sensory, and communicative function due to nervous system issues, as well as patient support and reintegration into their social and professional contexts [16].

## Case presentation

Patient information

A 65-year-old male, an auto-rickshaw driver, was seen following a road traffic accident (RTA) with a pedestrian early in the morning. The patient was found unconscious at the site of the incident and taken to hospital. He had a history of trauma, nasal bleeding, and a right ear bleed. There was no history of fever, vomiting, or seizures. After examination, the patient’s Glasgow Coma Scale (GCS) score was 3/15 (E1V1M1). The patient was seen within minutes for a neurology assessment, and a computed tomography (CT) scan of the skull revealed SDH and SAH, and the patient was managed conservatively. Antibiotics, antiemetics, analgesics, antifibrinolytics, and antacids are among the medications administered during the patient’s conservative care, and the patient was on 6 liters of oxygen via a facemask.

Clinical findings

Before the commencement of the physical examination, the patient provided his informed consent and subsequently underwent the assessment. Throughout the examination, the patient displayed a cooperative demeanor and demonstrated a clear orientation to place, person, and time, with a GCS score of 8 out of 15. The patient was positioned in a supine state and remained hemodynamically stable throughout the assessment. During the neurological examination, a reduction in tone was observed in the left upper and lower limbs (as depicted in Table 1). All deep tendon reflexes were noted to be diminished (as shown in Table 2). The voluntary grading scale (VCG) revealed a half range of motion (ROM) in synergy on the right side, while the left side displayed the initiation of movement (as detailed in Table 3). In addition, Babinski’s sign was noted to be positive. Based on these findings, a clinical diagnosis of hemiparesis on the left side was made, rendering the patient unable to stand or walk. Furthermore, the functional independence measure (FIM) indicated that the patient required maximal assistance with basic activities of daily living (ADLs). Consequently, the patient was referred for neurophysiotherapy.

**Table 1:** Muscle tone 0: flaccidity, 1+: hypotonia, 2+: normal response, 3+: mild to moderate hypertonia, 4+: severe hypertonia

Pre-rehabilitation			Post-rehabilitation		
Side	Lower limb	Upper limb	Side	Lower limb	Upper limb
Right	2+	2+	Right	2+	2+
Left	1+	1+	Left	2+	2+

**Table 2:** Deep tendon reflexes (DTR) +: diminished reflex, ++: normal reflex, +++: brisk reflex, ++++: exaggerated reflex

DTR	Pre-rehabilitation		Post-rehabilitation	
Reflexs	Right	Left	Right	Left
Biceps jerk	+	+	++	++
Triceps jerk	+	+	++	++
Knee jerk	+	+	++	++
Ankle jerk	+	+	++	++
Plantar jerk	Flexor response	Extensor response	Flexor response	Flexor response

**Table 3:** Voluntary grading scale VCG: Voluntary control grading Grade 1: flicker of contraction present or the initiation of movement; Grade 2: half range of motion (ROM) in synergy or abnormal pattern; Grade 4: initial half range is performed in isolation and latter half in pattern; Grade 5: full ROM in isolation but goes into pattern when resistance is offered; Grade 6: full ROM isolation against resistance

VCG	Pre-rehabilitation		Post-rehabilitation	
Limb	Right	Left	Left	Left
Upper limb	Grade 2	Grade 1	Grade 5	Grade 4
Lower limb	Grade 2	Grade 1	Grade 5	Grade 4

Diagnostic assessment

**Figure 1:**
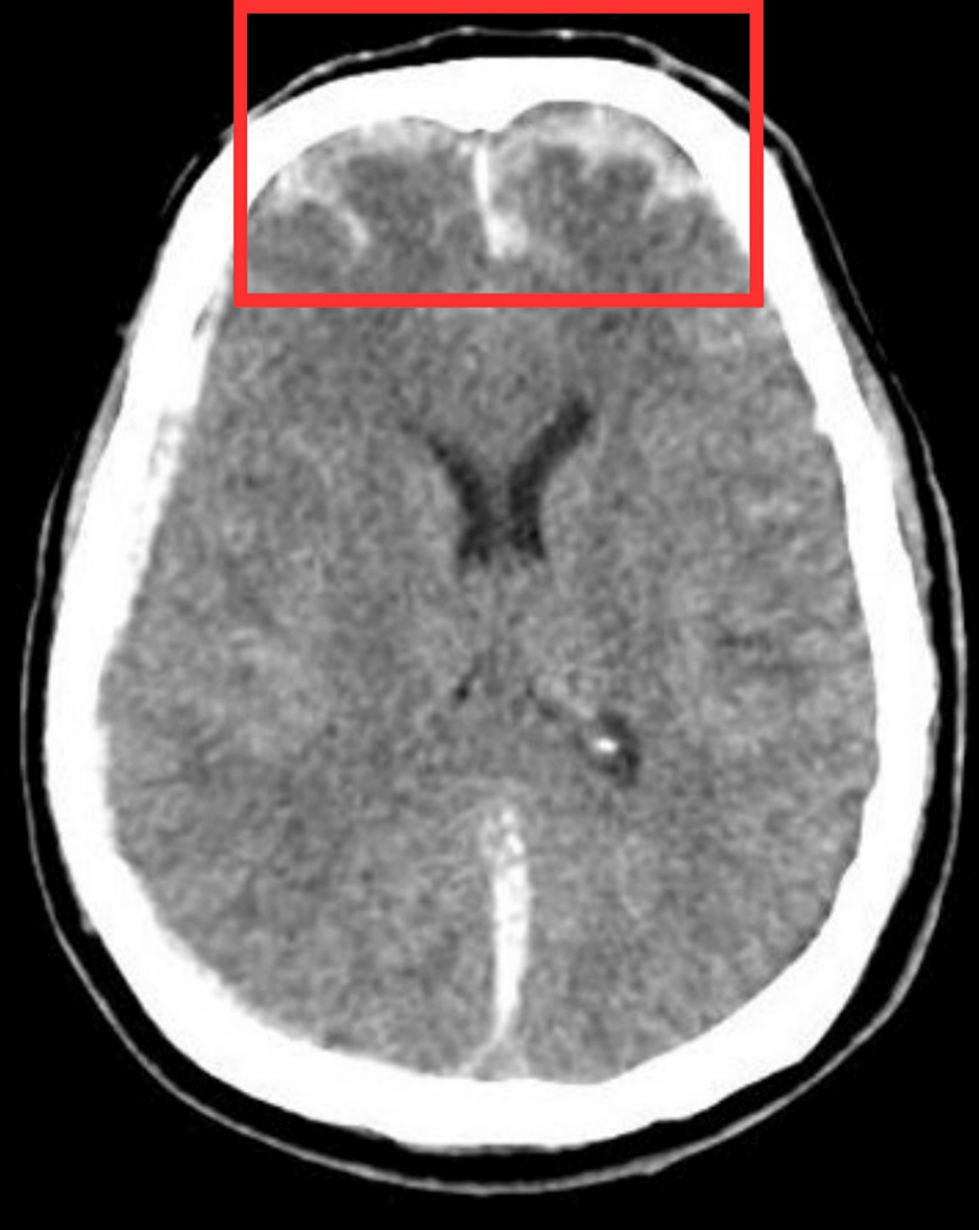
Computed tomography scan of the brain. The red rectangle shows a subdural hematoma on the left side.

A CT scan revealed a fracture in the occipital bone accompanied by swelling. In addition, fractures were observed in the right temporal bone along the petrous bone and mastoid process. Furthermore, hyperdense SDH was detected along the interhemispheric fissure and tentorial lining. A hyperdense hemorrhagic contusion was also observed in the bilateral temporal and frontal regions. Focal SAH was identified along the bilateral frontoparietal and Parisian fissure. Mild mucosal thickening was noted on both sides of the ethmoid and maxillary sinuses (Figure 1).

Physiotherapy intervention

**Figure 2:**
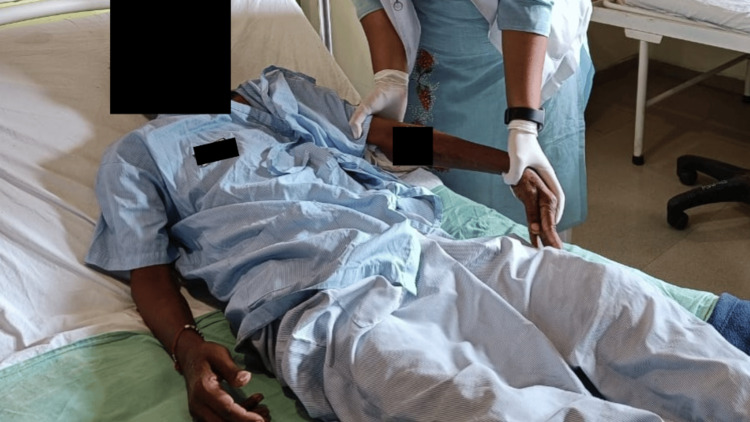
Proprioceptive neuromuscular facilitation

**Figure 3:**
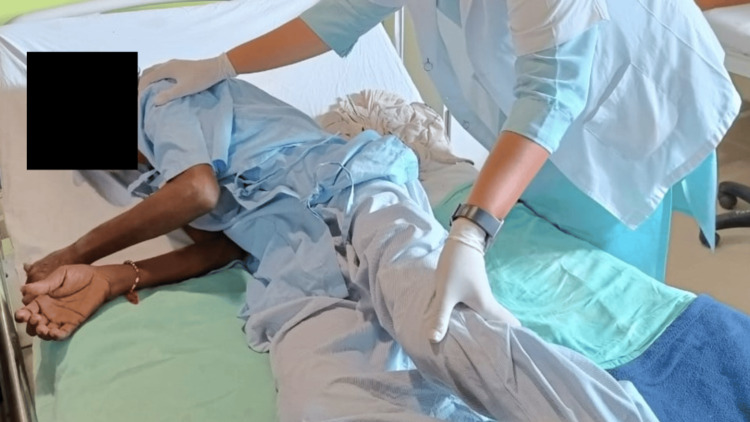
Bed mobility exercise

A tailored rehabilitation protocol was developed for the patient to address their needs. The patient underwent physiotherapy for four weeks, with sessions occurring five days a week. Table 4 outlines the details of the physical therapy protocol that was implemented. In Figure 2, the therapist can be seen administering upper limb proprioceptive neuromuscular facilitation (PNF) therapy to the patient. In addition, Figure 3 depicts the therapist assisting the patient with bed mobility exercises.

**Table 4:** Rehablitation protocol PNF: proprioceptive neuromuscular facilitation, reps: repetitions, ROM: range of motion, PROM: passive range of motion, DVT: deep vein thrombosis, D: diagonal

Goals	Therapeutic interventions	Treatment protocol	Dosage
To normalize the tone of muscles	Rood’s approach was given	Joint approximation to left upper and lower limb joints	10 reps
	PNF to left upper and lower limb	Rhythmic initiation technique was given to correct the muscle imbalance and restore the patient’s ability to perform effective coordinated movement. A D1 flexion-extension pattern was used.	10 reps with 2 sets
		Progression was given with PNF in combinations of isotonic to slow reversal.	10 reps with 2 sets
To enhance mobility	ROM	PROM exercises were commenced.	10 reps twice a day
To prevent DVT	Ankle exercises	Passive ankle pumps along with heel slides and DVT stockings	20 reps three times a day
Bronchial hygiene	Chest physiotherapy	Positioning, manual chest techniques (percussion and vibrations), and suctioning was given.	Change in position every 2-3 hours and manual techniques for 10 reps thrice a day
Prevention of contracture	Prolonged splinting and positioning	Sustained stretch to tendo-achilles	Relaxation for 10 seconds and hold time for 30-40 seconds
To prevent bed sores and to improve bed mobility	Segmental rolling techniques	Rolling facilitation that included rolling from supine to side-lying using upper extremity momentum and crossing the ankle pattern. Transition training from supine to sitting that inluded prone on the elbows from prone on elbows to long sitting, use elbow walking, supine on elbows.	Change in position in every 2-3 hours

Follow-up and outcome measures

During the fourth week of rehabilitation, the patient was subjected to a follow-up assessment, wherein outcome measures were estimated before and after the rehabilitation process. Table 5 shows the detailed information on these measures.

**Table 5:** Outcome measures CRS: Coma Remission Scale, GCS: Glasgow coma scale, FIM: Functional Independence Measure, MMSE: Mini-Mental State Examination

Outcome measures	Pre-rehabilitation	Post-rehabilitation
GCS	8/15	12/15
FIM	35/126	102/126
CRS	8/23	18/23
MMSE	18/30	26/30

## Discussion

The incidence of SAH commonly occurs at the rate of 10-20 per 100,000 individuals, with the average age of patients being 50 years old [17]. Based on the study, early mobilization was established as feasible after proper safety screening, and almost all the participants could achieve higher mobility or sit at the edge of their bed at least once during their physiotherapy period. In addition, half of the physiotherapy interventions allowed for mobilization. However, neurological factors, such as moderate sleepiness or recent or impending neurological surgery, often hinder mobilization. It was found that most of the participants who could be mobilized advanced to walking four to five days after admission, and all of them had crossed the mobility milestone of sitting on the side of the bed. Unfavorable incidents linked to mobilization were few, fleeting, and self-limiting [18].

Saciri et al. (2002) conducted a study revealing significant variations in the severity of sickness, medical and surgical care, quantity of physiotherapy, length of hospital stay, and place of release among patients with acute SAH or SDH [19]. According to Karic et al. (2015), patients with non-traumatic SAH can benefit from an early mobility program emphasizing upright sitting, standing, walking, functional training, and therapeutic supine activities [20]. Therefore, early mobilization can be considered safe and practical for such patients.

This care report pertains to a patient with concurrent subdural and subarachnoid hematoma. Neurorehabilitation is a multifaceted field encompassing patient support, reintegration into social and professional contexts, and early rehabilitation of patients with compromised motor, cognitive, sensory, and communicative functions caused by nervous system disorders. The patient was subjected to a rhythmic initiation technique to correct muscle imbalances and restore the patient’s ability to perform effective coordinated movement. The primary goal of physical therapy was to enhance muscle mobility. To prevent contractures, prolonged splinting and positioning were administered. Upon admission to the hospital, the patient scored 18 on the Mini-Mental Scale Examination but increased to 26 following therapy sessions. The patient experienced cognitive impairment, but as his cognition improved, his conscious level also increased.

## Conclusions

This report provides evidence that patients with cerebral hemorrhage exhibit better prognoses when they engage in prompt mobilization and adhere to recommended physical therapy regimens. In addition, their quality of life (QOL) improves. The physiotherapeutic interventions employed in this case were highly effective, with a reasonable recovery rate that improved the patient’s QOL, muscle strength, and mobility. Patients can achieve a higher QOL when they receive physiotherapy tailored to their individual needs and objectives, focusing on enhancing function, engagement, and symptom management.
